# Expression of *PLCZ1* mRNA in spermatozoa of Criollo and European bulls in a low-input system

**DOI:** 10.1590/1984-3143-AR2023-0053

**Published:** 2024-01-05

**Authors:** Luis Miguel Ronquillo-Roacho, Felipe Alonso Rodriguez-Almeida, Javier Antillón-Ruiz, Francisco Joel Jahuey-Martinez, Joel Domínguez-Viveros, José Alfredo Martínez-Quintana

**Affiliations:** 1 Facultad de Zootecnia y Ecología, Universidad Autónoma de Chihuahua, Chihuahua, Chihuahua, México

**Keywords:** gene expression, fertility, indigenous breeds, semen

## Abstract

Sperm motility and kinematics analysis are important to predict bull fertility. However, there are other molecules in the sperm with the ability to improve the pregnancy rate. For example, PLCZ1 is a sperm protein that plays a unique role in the activation of the zygote and is important for the survival of the embryo. The objective of this work was to compare the expression of *PLCZ1* mRNA in sperm cells of Chihuahuan Criollo and European bulls in the winter and summer seasons, under a low-input system. Six (3.33 ± 0.43 years old) bulls (three Criollo, three European) were used. Gross and individual motility were measured in semen obtained by electrostimulation. The cell pack was pelletized by centrifugation and stored in liquid nitrogen. The sperm cells were purified and total RNA was extracted. cDNA was synthesized to perform qPCR and measure the relative level of *PLCZ1* transcripts in each bull. There were no differences in individual motility, however, gross motility was lower (P < 0.05) in Criollo bulls, both in the winter (71.1 ± 2.8 vs. 76.6 ± 2.8%) and in the summer season (58.9 ± 2.8 vs. 77.7 ± 2.8%). *PLCZ1* expression was 5.3 times higher (P < 0.05) in winter than in summer (5.09 ± 1.09 vs 0.959 ± 1.09). No difference (P>0.05) was found in the expression levels of PLCZ1 between both breeds (4.36 ± 1.09 vs 1.69 ± 1.09), for Criollo and European, respectively. Although the animals presented seminal motility within the recommended limits for insemination, the expression levels of PLCZ1 vary depending on the time of the year and this might impact the rate of successful pregnancies. Therefore, it is important to complement conventional analysis of seminal quality with molecular characteristics.

## Introduction

Chihuahuan Criollo cattle is one of the 33 criollo bovine biotypes that exist in the American continent (De Alba Martínez, 2011). The great adaptability of these cattle allowed them, since their arrival to the continent, to colonize a great variety of regions and environments, including desertic and semi-desertic regions of northern Mexico and southern USA (Armstrong et al., 2022). Chihuahuan Criollo cattle (commonly named Rarámuri cattle, due to the indigenous ethnic group of the region in which this biotype is located; (De Alba Martínez, 2011)) is considered an ideal resource to produce in rough ecosystems, with low rainfall and scarcity of forage resources (Anderson et al., 2015), representing a potential genetic reservoir to achieve the sustainability of livestock production systems, even more so, given the problems caused by the advance of accelerated climate change (Holechek et al., 2020). With the aim of better introducing Rarámuri Criollo in production systems, it is important to study their physiological and molecular characteristics.

Among the great advantages that have been documented about Chihuahuan Criollo cattle, search capacity and diversification of its diet stand out. These cattle tend to browse arboreal and shrubby vegetation, consume fruits and a variety of herbaceous plants and grasses that specialized cattle do not normally consume (Anderson et al., 2015; Armstrong et al., 2022). It is known that the type of diet consumed by animals results in changes in gene expression in different tissues and cells, including sperm (Shibata et al., 2014; Krishnaiah et al., 2019).

Raramuri Criollo bulls have good semen quality throughout the year and high testosterone concentrations (Quezada-Casasola et al., 2018), however, ensuring semen quality measured in the conventional way does not guarantee embryo viability (Franco et al., 2020). Therefore, in recent years, in addition to the conventional analyses of seminal quality and viability, important biochemical and physiological indicators for achieving successful pregnancy are being implemented (Atabay et al., 2019; Krishnaiah et al., 2019; Franco et al., 2020). These analyses might include molecular biomarkers such as: metabolites, enzymes and RNAs (Le Blévec et al., 2020).

At the time of gamete fusion, a series of intracellular Ca^2+^ oscillations occur, which are essential for the oocyte to complete meiosis and for the activation of the zygote to occur. The release of Ca^2+^ is induced by an enzyme called PLCZ1 (Kashir et al., 2018). *PLCZ1* mRNA is specific to spermatids (Ramadan et al., 2012) and a positive relationship has been found between the remnants of this mRNA and the amount of PLCZ1 protein within the spermatozoon, in addition to a possible further functionality (Aghajanpour et al., 2011). PLCZ1 mRNA levels are higher in more fertile individuals, compared to the less fertile ones in human males (Aghajanpour et al., 2011), buffaloes (Atabay et al., 2019) and Holstein bulls (Kasimanickam et al., 2012). The objective of this study was to evaluate whether, under a low-input production system, the expression of PLCZ1 mRNA in sperm cells varies between breeds (Raramuri Criollo and Europeans) and times of the year (winter, summer).

## Methods

### Study site and animals

All the experiments were carried out in accordance with the UK Animals (Scientific Procedures) Act 1986. The study was reviewed and approved by the Institutional Bioethics Committee of the Facultad de Zootecnia y Ecología at the Universidad Autónoma de Chihuahua, Mexico (Decision No. P/302/2017).

The bulls were at the Teseachic Experimental Ranch owned by the Universidad Autónoma de Chihuahua (UACH), located to the east of the Sierra Madre Occidental (28°4' N; 107° 25' E), in Chihuahua, Mexico. The climate at that location is characterized by cold winters and hot summers. The elevation varies between 1,900 and 2,800 m, with gentle and broken slopes. The bulls remained grazing without food supplementation in a common pasture with an extension of 80 ha. A total of six healthy bulls (3.33 ± 0.43 years old) were used, three Chihuahuan Criollo and three European (2 Hereford, 1 Angus).

The expression analyses were carried out in the Molecular Biology laboratory of the Facultad de Zootecnia y Ecología of the UACH.

### Collection and seminal evaluation

Semen was collected by electro-stimulation with a 60-mm diameter bipolar electrode device. The collector was connected by a plastic cone to a sterile conical tube (15 mL). The macroscopic and microscopic characteristics of the semen were immediately evaluated. Gross and individual motility were determined subjectively by the same technician, with a microscope (Velab™) at 10 and 40 X, respectively. Subsequently, 2 mL of the ejaculate were centrifuged (Prism mini™) at 4,000 rpm for 5 min to separate and then discard the seminal fluid. The cell pack was immersed in liquid nitrogen to be transported to the laboratory and then stored at -80°C until its use in subsequent steps of spermatozoa purification and RNA extraction. The samplings were carried out in triplicate with an interval of 5 d between each one, in each season (winter and summer).

### Sperm cell purification

In order to eliminate impurities and somatic cells from the seminal fluid, spermatozoa were purified using the commercial BoviPure^TM^ System (Nidacon) as reported by Parthipan et al. (2015). Briefly, the pellet was resuspended in 1 mL of PBS and the suspension was then transferred as a second phase onto 4 mL of BoviPure^TM^ in a 15 mL tube. The spermatozoa were centrifuged at 1280 rpm/20 min and the pellet was washed twice with 10 mL of PBS at 2390 rpm/5 min.

### Total RNA extraction

RNA was extracted from approximately 60 million sperm cells from each of the 36 ejaculates corresponding to three replicates from each of six bulls at two seasons, using the Direct-Zol^TM^ RNA MiniPrep Plus (ZYMO) Kit. The 60 million cells resuspended in PBS were centrifuged at 4,000 rpm and the supernatant discarded. The cell pellet was resuspended in 1 mL of Trizol (TRI Reagent^®^, Sigma Aldrich), incubated at 65°C for 30 min, and mixed vigorously for 15 s. Subsequently, the manufacturer's instructions were followed. RNA quantification was performed on a NanoDrop^TM^ 2000 spectrophotometer (Thermo Fisher Scientific).

### cDNA synthesis

cDNA was synthesized separately for each bull, pooling together the three replicates of RNA for each time of the year. The synthesis was done from 250 ng of total RNA using the PrimeScript^TM^ RT reagent Kit with gDNA Eraser (Takara), following the manufacturer's instructions.

### Quality control of sperm RNA

To verify the purity of sperm RNA, real-time PCR was performed for three specific genes: CDH1, which would only be expressed in somatic cells; KIT, which would only be expressed in germ cells; and PTPRC which would only be expressed in leukocytes. In addition, to rule out the presence of genomic DNA (gDNA), a PCR was performed for the protamine gene (PRM1) that generates a longer amplicon from gDNA than from cDNA. The PCR primers and conditions used were as reported by Selvaraju et al. (2017).

### Oligonucleotide design

Oligonucleotides for the bovine *PLCZ1* gene and for the reference *β-actin* (*β-Act*) gene were designed using the NCBI primer-BLAST program, according to Ye et al. (2012), based on the reference sequences of the genbank NM_001011680.3 and AY141970.1, respectively. Oligonucleotides for *PLCZ1* were: PLCZ1-F (5´ AACTTAGCCTCCAGAACAGCC 3´) and PLCZ1-R (5´ CGCTTGGCAAGAAAGGGATTC 3´), and for *β-Act* gene: BBactin-F (5´ CGGGACCTGACGGACTACCT 3´) and BBactin-R (5´ TGTCACGGACGATTTCCGCTC 3´).

### Quantification of the expression of the *PLCZ1* gene relative to β-Act

The real-time qPCR reaction was performed in a thermocycler (AB Applied Biosystems StepOne Real-Time PCR System™) in a final volume of 10 μL, containing: 5 μL of 2X powerUp^TM^ SYBER™ Green Master Mix, 900 nM of PLCZ1 oligonucleotides or 500 nM of β- Act oligonucleotides, 12.5 ng of cDNA from total RNA and nuclease-free water. Each qPCR run was accompanied by a standard curve composed of six points, with a dynamic range of 12.5 to 5.14 x 10^-2^, corresponding to total RNA and generated in 1:3 serial dilutions of the most concentrated point.

PCR conditions for PLCZ1 were as follows: one step at 50°C for 2 min and 95°C for 2 min, followed by 40 cycles of 15 s at 95°C, 15 s at 53°C, and 40 s at 72°C. For β-Act only the alignment temperature changed to 58°C. Both PCRs were run with a single measurement of the fluorescence signal taken in the extension step. Additionally, a final dissociation curve performed from 60°C to 95°C, with 0.3°C increases every 5 s. The expression of *PLCZ1* is reported in relation to the expression of β-Act with the method of 2^-ΔCT^ (Schmittgen and Livak, 2008).

### Statistical analysis

The statistical analysis for the variables of individual motility, gross motility and the expression of *PLCZ1*, a mixed model was adjusted that included the fixed effects of season, breed and their interaction, and the bull as a random effect, it was analyzed with the PROC MIXED procedure from SAS software, version 9.2; SAS Institute, Inc., Copyright © 1999).

## Results

As shown in Table 1, no difference was found in the percentage of individual motility (IM) between breed or season; however, in gross motility (GM) there was a significant difference (P<0.05) by breed, being lower in Criollo animals than in European ones. In the interaction of breed by season, a numerical difference is observed, although not statistically significant (P=0.06), with the GM being slightly lower in Criollo bulls in the winter season, and this difference increased during the summer.

**Table 1:** Means ± standard error of individual and gross motility of Raramuri Criollo and European bovine semen in winter and summer.

**Motility (%)**	**Winter**		**Summer**		**S.E.**
	**European**	**Criollo**	**European**	**Criollo**	
Individual	78.8	72.2	76.6	64.4	± 4.3
Gross	76.6^a^	71.1^b^	77.7^a^	58.9^b^	± 2.8

^a^, ^b^, Different letters indicate statistical difference (P<0.05) between columns; S.E.: standard error.

**Figure 1:**
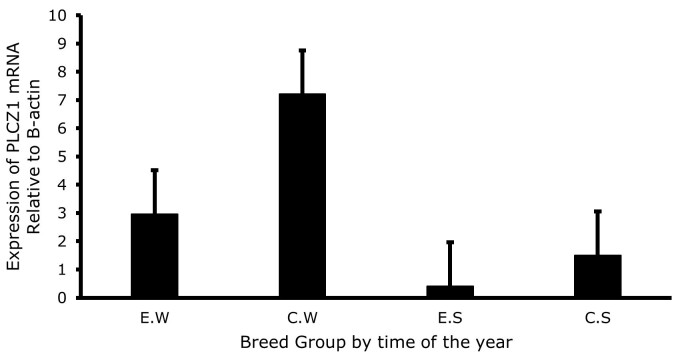
Expression level of *PLCZ1* mRNA relative to β-actin in spermatozoa from Raramuri Criollo and European bulls, by season of the year (E.W = European Winter; C.W = Criollo Winter; E.S = European Summer; C.S = Criollo Summer). The bars show the means ± standard error calculated with the 2^-ΔCT^ method, n = 3.

**Figure 2:**
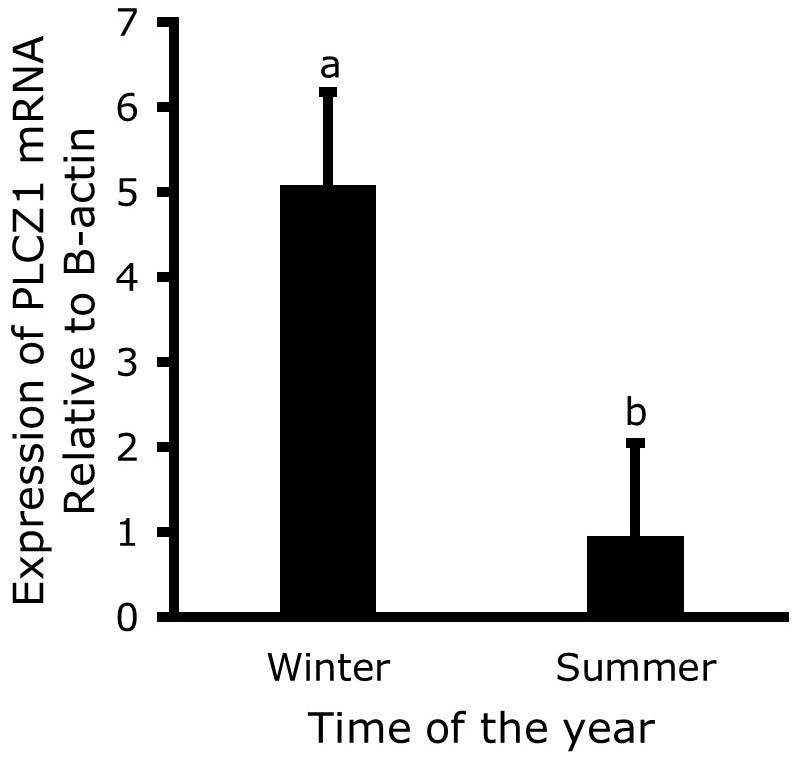
*PLCZ1* mRNA expression level relative to β-actin in bull spermatozoa, in the winter and summer seasons. The bars show the means ± standard error calculated with the 2^-ΔCT^ method, n=6.

**Figure 3:**
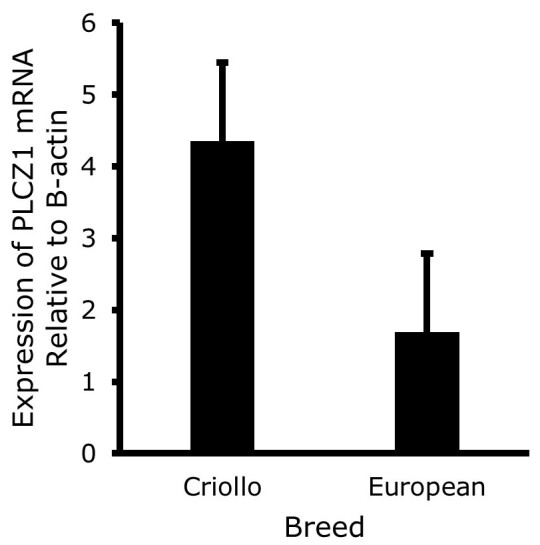
*PLCZ1* mRNA expression level relative to β-actin in Criollo and European bull spermatozoa. The bars show the means ± standard error calculated with the 2^-ΔCT^ method, n=6.

The expression of *PLCZ1* in sperm cells of Raramuri Criollo and European bulls in the two seasons of the year are presented in Figure 1, with no breed effect and season interaction was found. However, as shown in Figure 2, the expression of *PLCZ1* was higher (P<0.05) in the winter season than in summer (5.09 ± 1.09 vs 0.959 ± 1.09). Also, a numerical —although no statistically significant (P=0.16)— difference was found between breeds, as shown in Figure 3.

## Discussion

The acceleration of global climate change has prompted the implementation of different strategies to reduce the negative effects of livestock production in extensive production systems. One of the strategies that has been strongly implemented in recent years is the use of breeds adapted to stressful conditions due to high temperatures, food shortages and low water availability. In this regard, Chihuahuan Criollo cattle (Rarámuri cattle) have shown great advantages over other breeds in arid and semi-arid regions of the southwestern United States and northern Mexico (Holechek et al., 2020). Therefore, it is important to study and characterize the physiology of this breed, which had been forgotten due to the introduction of specialized breeds (Anderson et al., 2015).

The semen of the bulls of both breeds presented good motility values in the tests; Criollo IM values in winter and summer (Table 1) were similar to those obtained by Villatoro-Salinas et al. (2016) in Criollo tropical milking bulls from Veracruz in cool season (68.3 ± 5.4%) and hot season (75.7 ± 5.4%). Quezada-Casasola et al. (2018) report that IM in Chihuahuan Criollo bulls remains constant throughout the year (Spring 80.7 ± 6, Summer 82.9 ± 6, Autumn 83.5 ± 6 and Winter 84.9 ± 6%), while that in bulls from European breeds, motility decreases during the hottest season (Spring 83.9 ± 9, Summer 62.3 ± 9, Autumn 58.4 ± 9 and Winter 85.15 ± 9%), however, the animals studied by these authors were housed and given a balanced diet.

Despite the fact that GM was lower (P<0.05) in Criollo bulls compared to European ones, it is important to mention that the values do reach the minimum levels considered by experts for both, natural mating in extensive systems and artificial insemination (Crespilho et al., 2012; Vélez-Castañeda et al., 2014). It is also important to mention that, consistently, at the time of sampling, the Chihuahuan Criollo showed a retraction of the penis, resulting in contact between the ejaculate and the foreskin. This creates the need to standardize a protocol for obtaining semen from this type of animal, ensuring good practices that prevent the deterioration of semen quality.

There is evidence that the PLCZ1 protein is essential to trigger oocyte activation (Ramadan et al., 2012; Kashir et al., 2018) and that the higher the levels of PLCZ1 in the spermatozoa, the higher the percentage of oocyte fertilization (Aghajanpour et al., 2011; Atabay et al., 2019). As observed in Fig. 2, a difference was found in the relative expression levels of PLCZ1 by season of the year, being 5.3 times higher in winter than in summer time. This difference can be attributed to environmental effects such as temperature and the nutritional level of the animals, since nutrition has a great impact on gene expression in all tissues, including sperm (Shibata et al., 2014; Krishnaiah et al., 2019). For example, vitamin and mineral supplementation has been reported to have a large effect on the expression of different genes (Lin et al., 2017; Krishnaiah et al., 2019). In a study in which the expression of different genes of reproductive interest in mouse spermatozoa were evaluated by real-time qPCR, the supplementation in mice with carob extract increased the expression of PLCZ1 by 0.36 and 0.33 times at 21 and 35 days of supplementation (ElAkhdar et al., 2020).

It has been reported that in mammalian spermatozoa there are different types of RNAs and other molecules that are delivered to the oocyte together with the paternal gDNA (Selvaraju et al., 2017; Le Blévec et al., 2020). For instance, Franco et al. (2020) demonstrated embryonic or fetal loses associated to some specific bulls as semen donators in an experiment where these animals had no differences in the conventional semen quality criteria for fertilization by artificial insemination. After inseminating a different number of cows per bull, they obtained a 52% pregnancy at 24 d post insemination. At 31 d of pregnancy there was an embryonic loss of 5.5% and at 60 d the number of pregnant cows decreased again from 46.5% to 39.8%. These embryonic or fetal losses were evident only in cows inseminated by certain bulls, indicating that spermatozoa may contain different types of RNA from certain genes involved with embryo survival during the most critical days. Therefore, the identification of molecular markers with an important effect on fertility and embryonic survival, in addition to the conventional evaluation of semen quality, could increase the pregnancy rate in production systems.

The expression of PLCZ1 mRNA *per* individual was different when evaluated in a large group of water buffaloes under the same environmental and management conditions, finding a positive correlation between PLCZ1 mRNA levels and fertility of the animals (Atabay et al., 2019). In our study, as shown in Figure 3, Chihuahuan Criollo bulls tended to have higher expression levels of PLCZ1 mRNA compared to bulls from European breeds, with gene expression levels varying according to the individual, the diet and the genotype. Regarding fertility, this is in line with the findings of Torell et al. (2023), who reported a calf yield of 91% in Rarámuri Criollo cattle and 85% in European cattle using a 30:1 and 16:1 cow-bull ratio, respectively, which suggests higher fertility rates for Criollo bulls. As mentioned in the introduction, Chihuahuan Criollo cattle have a different distribution and grazing habits than European ones, so their level of nutrition could change according to this differences in behavior. It is interesting that despite the fact that the gross motility of the Criollo semen was lower than that of the semen of European bulls, the expression levels of PLCZ1 mRNA tended to be higher in Criollo in the two seasons evaluated. These results suggest the need to measure the levels of PLCZ1 mRNA in the sperm of a larger number of animals and search for the association with to the successful pregnancy rate in cows.

## Conclusion

The mRNA PLCZ1 expression level in the sperm cells is affected by the time of the year, suggesting that food quality and food availability can influence gene expression in these cells.
